# Efficient Enzymatic Routes for the Synthesis of New Eight-Membered Cyclic β-Amino Acid and β-Lactam Enantiomers

**DOI:** 10.3390/molecules22122211

**Published:** 2017-12-13

**Authors:** Enikő Forró, Loránd Kiss, Judit Árva, Ferenc Fülöp

**Affiliations:** 1 Institute of Pharmaceutical Chemistry, University of Szeged, H-6720 Szeged, Eötvös u. 6, Hungary; kiss.lorand@pharm.u-szeged.hu (L.K.); Arva.Judit@pharm.u-szeged.hu (J.A.); 2 Research Group of Stereochemistry of the Hungarian Academy of Sciences, University of Szeged, H-6720 Szeged, Eötvös u. 6, Hungary

**Keywords:** anatoxin-*a*, β-Amino acid, enzyme catalysis, β-Lactam, traceless activating group

## Abstract

Efficient enzymatic resolutions are reported for the preparation of new eight-membered ring-fused enantiomeric β-amino acids [(1*R*,2*S*)-**9** and (1*S*,2*R*)-**9**] and β-lactams [(1*S*,8*R*)-**3**, (1*R*,8*S*)-**3** (1*S*,8*R*)-**4** and (1*R*,8*S*)-**7**], through asymmetric acylation of (±)-**4** (*E* > 100) or enantioselective hydrolysis (*E* > 200) of the corresponding inactivated (±)-**3** or activated (±)-**4** β-lactams, catalyzed by PSIM or CAL-B in an organic solvent. CAL-B-catalyzed ring cleavage of (±)-**6** (*E* > 200) resulted in the unreacted (1*S*,8*R*)-**6**, potential intermediate for the synthesis of enantiomeric anatoxin-*a*. The best strategies, in view of *E*, reaction rate and product yields, which underline the importance of substrate engineering, are highlighted.

## 1. Introduction

Chiral β-amino acids and β-lactams are important compounds because of their pharmacological properties and their use in diverse syntheses. For example, (1*R*,2*S*)-2-aminocyclopentanecarboxylic acid (cispentacin) [1], the simplest, naturally occurring carbocyclic β-amino acid, is an antifungal antibiotic, but can also be found in the structures of some natural products, e.g., amipurimycin [2]. Its methylene derivative, (1*R*,2*S*)-2-amino-4-methylenecyclopentanecarboxylic acid (icofungipen, PLD-118) [3], in turn, is active in vitro against *Candida species*. Enantiomeric eight-membered ring-fused β-lactams (1*R*,8*S*)- and (1*S*,8*R*)-9-azabicyclo[6.2.0]dec-4-en-10-one are potential key intermediates [4] in the syntheses of anatoxin-*a* [5]. It is a neurotoxic alkaloid, one of the most toxic of the cyanobacterial toxins, but a potent and stereospecific agonist at nicotinic acetylcholine receptors [5,6,7]. A relatively large number of publications deal with its isolation from strains of *Anabaena flos aqua*, a freshwater blue-green alga, but synthetic methods [8,9,10], also for the preparation of new anatoxin-*a* homologues [11] have also been described. Cyclic β-amino acids can serve as building blocks for the synthesis of modified peptides with increased activity and stability [12] and with well-defined three-dimensional structures (foldamers) (e.g., β-peptides with possible antibiotic activity) similar to those of natural peptides [13,14]. Additionally, the alkene functionality in molecules is amenable to a range of transformations. Cyclic β-amino acids can also be used in heterocyclic [15,16] and combinatorial [17] chemistry.

In addition to conventional resolution methods for the preparation of enantiomeric β-amino acids and β-lactams, enzymatic strategies have also been described [18,19,20]. Our research group has also devised a number of efficient enzymatic kinetic and sequential kinetic resolution processes (acylations, deacylations and hydrolyses). Most of these methods have been reviewed [21,22,23].

**Figure 1:**
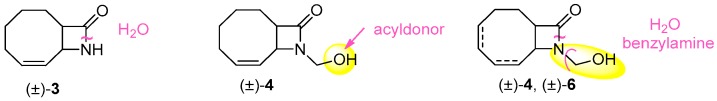
Substrates (±)-**3**, (±)-**4** and (±)-**6** in the enzymatic strategies planned.

A primary aim of this work was to devise adequate enzymatic strategies for the preparation of valuable new enantiomeric eight-membered carbocyclic β-lactam and β-amino acid derivatives. In view of earlier results on enzymatic acylation of *N*-hydroxymethyl eight-membered carbocyclic β-lactams [4,24], we first planned to carry out enzymatic acylation of *N*-hydroymethyl-9-azabicyclo[6.2.0]dec-6-en-10-one [(±)-**4**] (Figure 1). The extensive investigations of the lipase-catalyzed ring cleavage of unactivated [25,26,27] and activated [28] β-lactams then suggested the possibility of the lipase-catalyzed enantioselective ring cleavage of racemic unactivated 9-azabicyclo[6.2.0]dec-6-en-10-one [(±)-**3**] and activated (±)-**4** and *N*-hydroxymethyl 9-azabicyclo[6.2.0]dec-4-en-10-one [(±)-**6**]. A systematic comparison of the efficiency of these methods, with regard to *E*, reaction rate and yield for the products, was also intended.

## 2. Results and Discussion

### 2.1. Synthesis of β-lactams (±)-***3***–(±)-***6***

**Scheme 1:**
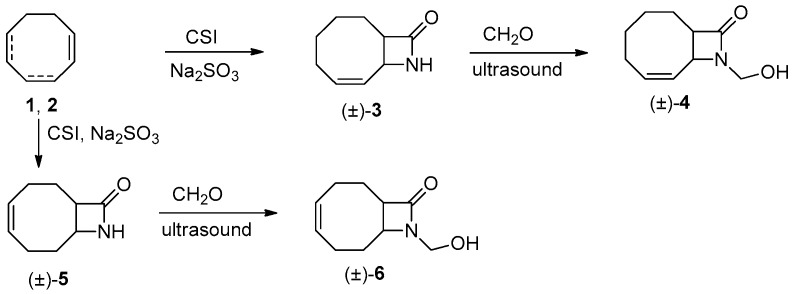
Synthesis of (±)-**3**–(±)-**6**.

Lactams (±)-**3** and (±)-**5** were synthesised from cyclooctadiene **1** or **2** by the addition of chlorosulfonyl isocyanate (CSI), according to a slightly modified literature procedure (Scheme 1) [29,30]. Then product lactams were reacted with paraformaldehyde under sonication to form *N*-hydroxymethyl β-lactams (±)-**4** and (±)-**6** [4].

### 2.2. Lipase-Catalyzed O-acylation of (±)-***4***

**Scheme 2:**
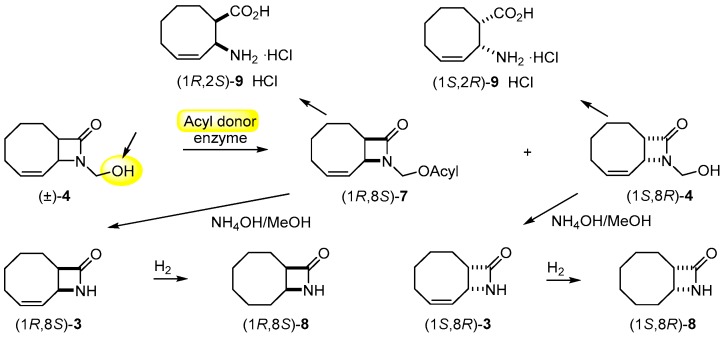
Lipase-catalyzed *O*-acylation of (±)-**4**.

On the basis of the earlier results on the enzymatic resolution of *N*-hydroxymethyl 9-azabicyclo[6.2.0]dec-4-en-10-one [4] and *N*-hydroxymethyl 9-azabicyclo[6.2.0]decane-4-en-10-one [24], first the acylation of (±)-**4** (Scheme 2) was carried out with vinyl butyrate (VB) in the presence of PSIM (*Burkholderia cepacia*) in diisopropyl ether (*i*Pr_2_O) at −15 °C (Table 1, entry 1).

When the acylation was performed at higher temperatures, the reaction rate increased with the concomitant decrease in *E* (entries 2 and 3 vs. 1). As the amount of VB was increased from 2 to 10 equiv., the reaction rate increased while *E* apparently decreased (entry 4 vs. 3). The addition of a catalytic amount of Et_3_N and Na_2_SO_4_ resulted in a clear increase in *E* (entry 5 vs. 4). 

To further increase the *E* values, acyl donors, such as 2,2,2-trifluoroethyl butyrate, vinyl acetate (VA), ethyl acetate (EtOAc) and acetic anhydride (Ac_2_O) were tested (entries 6–9). Unfortunately, none of the acyl donors tested exerted any beneficial influence on the reaction course. Several solvents have also been tested. When *i*Pr_2_O was replaced by *t*BuOMe, practically no change was observed in the reaction course (entry 15 vs. 4). The same high *E* but somewhat lower reaction rate was observed in acetone (entry 17), while the best *E* and fastest reaction were detected in toluene (entry 16). Finally the environmentally less harmful iPr_2_O was selected as solvent.

In addition to lipase PSIM, several enzymes, such as lipase AK (*Pseudomonas fluorescens*), lipase AY (*Candida rugosa*), CAL-A (lipase A from *Candida antarctica*), CAL-B (lipase B from *Candida antarctica*) and PPL (porcine pancreatic lipase) have been investigated (entries 10–14). However, in terms of *E* and reaction rate, none of them proved to be better than PSIM. 

### 2.3. Lipase-Catalyzed Ring Cleavage of (±)-***3*–**(±)-***6***

**Scheme 3:**
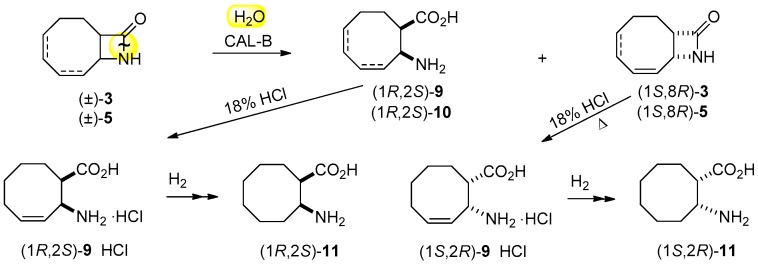
Lipase-catalyzed ring cleavage of (±)-**3** and (±)-**5**.

In view of the results on the lipase-catalyzed ring cleavage of carbocyclic β-lactams [25,26,27], the ring opening of (±)-**3** was attempted with 1 equiv. of H_2_O in the presence of CAL-B (lipase B from *Candida antarctica*) in *i*Pr_2_O at 60 °C (Scheme 3; Table 2, entry 1). When the ring cleavage of the regioisomeric 9-azabicyclo[6.2.0]dec-4-en-10-one [(±)-**5**] was performed under the same conditions, a similar fast reaction and the same high *ee* values (>98%) were observed (entry 2).

**Scheme 4:**
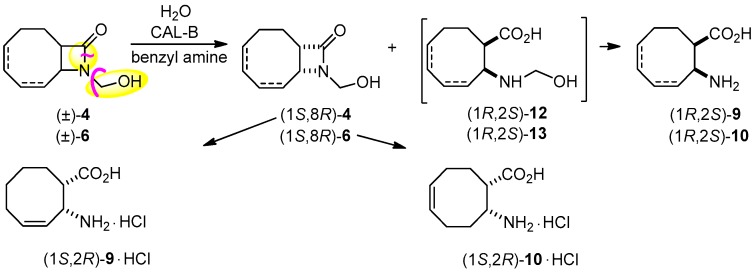
Lipase-catalyzed two-step transformation of (±)-**4** and (±)-**6**.

In further studies, we have probed our very recent results found about the ring cleavage of specially activated lactams [28], where the activating group underwent to a traceless, in situ degradation. Accordingly, the ring cleavage of (±)-**4** was attempted with H_2_O in the presence of CAL-B and benzylamine to capture formaldehyde in *i*Pr_2_O at 60 °C (Scheme 4, Table 2, entry 3). Excellent *ee* (>99%) characterized formed amino acid (1*R*,2*S*)-**9** at a conversion close to 50%. The ring cleavage of regioisomeric (±)-**6** was also carried out under the same conditions (entry 4), and the same high *ee* (>98%) for amino acid (1*R*,2*S*)-**10** and unreacted lactam (1*S*,8*R*)-**6**, potential intermediate in the synthesis of enantiomeric anatoxin-*a* was observed. In good accordance with the earlier observation that the hydroxymethyl group activates the ring cleavage of lactams, racemic **4** and **6** underwent ring cleavage much faster than their corresponding inactivated counterparts (entry 3 vs. 1 and 4 vs. 2).

On the basis of the preliminary results, preparative-scale resolutions of racemic **3**, **4** and **6** were performed under the optimized conditions (footnotes to Table 3). The results are reported in Table 3 and Experimental Section.

### 2.4. Further Transformations

Hydrolysis of enantiomeric **3**, **4**, **6** and **7** with 18% aqueous HCl (Scheme 2, Scheme 3 and Scheme 4) gave the corresponding hydrochloride salts **9**·HCl and **10**·HCl (*ee* > 97%), while treatment of enantiomeric **4** and **7** with NH_4_OH/MeOH (Scheme 2) resulted in β-lactams (1*R*,8*S*)-**3** and (1*S*,8*R*)-**3** (*ee* ≥ 95%). Catalytic reduction of enantiomeric lactams **3** and amino acids **9** with H_2_ or cyclohexene as a hydrogen donor and using palladium-on-carbon furnished saturated lactams (1*R*,8*S*)-**8** and (1*S*,8*R*)-**8**, and amino acids (1*R*,2*S*)-**11** and (1*S*,2*R*)-**11**, respectively (Scheme 2 and Scheme 3), without a drop in *ee* (>96%). Physical data on the enantiomers prepared are reported in Table 4 and Experimental Section.

### 2.5. Absolute Configurations

Absolute configurations were determined by comparing the [*α*] values of the enantiomeric compounds with literature data. Specifically, [*α*] values of enantiomeric compounds **8** (Table 4, entries 3 and 4), obtained from enantiomeric compounds **4** and **7** (Scheme 2), were compared with the literature data of (1*S*,8*R*)-**9**-azabicyclo[6.2.0]decan-10-one {${\left[\alpha \right]}_{D}^{25}$ = −18 (*c* = 0.5; CHCl_3_)} [25]. In a similar way, the [*α*] values of enantiomeric compounds **11** (Table 4, entries 9 and 10), obtained from enantiomeric compounds **9**·HCl (Scheme 3), were compared with the literature data for (1*R*,2*S*)-2-aminocyclooctane-1-carboxylic acid {${\left[\alpha \right]}_{D}^{25}$ = +17.8 (*c* = 0.4; H_2_O)} [25]. Thus, the (1*R*,8*S*) configuration is determined for ester **9** and the corresponding lactam (**3**), and the (1*S*,8*R*) configuration is assigned to unreacted alcohol **4** and the corresponding lactam (**3**). The CAL-B-catalyzed ring cleavage of either inactivated lactams **3** and **5** or activated lactams **4** and **6** afforded the corresponding amino acids with (1*R*,2*S*) absolute configuration I.

## 3. Experimental Section

### 3.1. Materials and Methods

CAL-B (lipase B from *Candida antarctica*), produced by the submerged fermentation of a genetically modified *Aspergillus oryzae* microorganism and adsorbed on a macroporous resin (Catalogue no. L4777), and porcine pancreatic lipase (PPL) were from Sigma-Aldrich (St. Louis, MO, USA). CAL-A (lipase A from *Candida antarctica*) was purchased from Roche Diagnostics Corporation. Lipase PSIM (*Burkholderia cepacia,* immobilized on diatomaceous earth) was a kind gift from Amano Enzyme Europe Ltd. (Suqian, China) Lipase AK (*Pseudomonas fluorescens*) was from Amano Pharmaceuticals, and lipase AY (*Candida rugosa*) was from Fluka. The solvents were of the highest analytical grade. Melting points were determined with a Kofler apparatus. NMR spectra were recorded on a Bruker DRX 400 spectrometer. Optical rotations were measured with a Perkin-Elmer 341 polarimeter. Elemental analyses were performed with a Perkin-Elmer CHNS-2400 Ser II Elemental Analyzer. The syntheses of racemic *N*-hydroxymethyl 9-azabicyclo[6.2.0]dec-6-en-10-one [(±)-**5**] and *N*-hydroxymethyl 9-azabicyclo[6.2.0]dec-4-en-10-one [(±)-**6**] were performed according to our earlier method [4].

### 3.2. Typical Small-Scale Enzymatic Experiments

Racemic β-lactam in an organic solvent (0.05 M solution, 1 mL) was added to the lipase tested (30 mg mL^−1^). The tested acyl donor (2 or 10 equiv.) in acylations or H_2_O (1 equiv.) in hydrolyses was added. The mixture was shaken at −15 °C, 2–3 °C, 30 °C or 60 °C. The progress of the reaction was followed by taking samples from the reaction mixture at intervals and analysing them by using a gas chromatograph equipped with a chiral column. The *ee* values for the unreacted β-lactam enantiomers were determined directly on a Chromopack Chiralsil-Dex CB column [retention times are given in min; 140 °C for 25 min → 190 °C (temperature rise 20 °C∙min^−1^; 140 kPa, (1*S*,8*R*)-**3**: 27.74 (antipode: 27.56); 140 °C for 7 min → 190 °C (temperature rise 10 °C min^−1^; 140 kPa, (1*S*,8*R*)-**5**: 12.55 (antipode: 12.12)] or on a Chirasil-L-Val column, [140 °C for 7 min → 190 °C (temperature rise 10 °C/min; 140 kPa), (1S,8R)-4: 14.39 (antipode: 14.61); (1S,8R)-6: 14.40 (antipode: 14.87); (1R,8S)-7: 14.67 (antipode: 14.36)]. The *ee* values for the β-amino acids produced were determined on a Chirasil-L-Val column, after double derivatization with (i) CH_2_N_2_ [31] (*caution! derivatization with diazomethane should be performed under a well*-*ventilated hood*); (ii) Ac_2_O in the presence of 4-dimethylaminopyridine and pyridine [120 °C for 7 min → 190 °C (temperature rise 10 °C min^−1^; 140 kPa, (1R,2S)-9: 12,41 (antipode: 12.95); (1R,2S)-10: 12.88 (antipode: 14.06)] and after double derivatization with (i) CH_2_N_2_ and (ii) (PropCO)_2_O in the presence of 4-dimethylaminopyridine and pyridine [140 °C for 10 min → 190 °C (temperature rise 10 °C min^−1^; 140 kPa; (1R,2S)-11: 17.58 (antipode: 17.78)]. 

### 3.3. Synthesis of Racemic 9-Azabicyclo[6.2.0]dec-6-en-10-one [(±)-***3***]

A solution of CSI (2.28 mL, 26.2 mmol) in CH_2_Cl_2_ (25 mL) was added dropwise to a stirred solution of 1,3-cyclooctadiene (3 mL, 26.2 mmol) in CH_2_Cl_2_ (25 mL) at 0 °C. The reaction mixture was stirred at room temperature for 21 h, and the resulting liquid was then added dropwise to a vigorously stirred solution of Na_2_SO_3_ (0.33 g) and K_2_CO_3_ (7.12 g) in H_2_O (50 mL). After stirring the mixture for 3.5 h, the combined organic layers were dried (Na_2_SO_4_) and, after filtration, concentrated. The resulting white solid, racemic **3** (3.41 g, 86%), was recrystallized from *i*Pr_2_O (m.p. 103–106 °C). 

The ^1^H-NMR (400 MHz, CDCl_3_, 25 °C, TMS) δ (ppm) data for (±)-**3**: 1.46–2.11 (8H, m, 4 × CH_2_), 3.34–3.38 (1H, dd, *J* = 5.34, 12.26 Hz, H-1), 4.57–4.58 (1H, m, H-8), 5.41–5.79 (2H, m, C*H*C*H*), 6.01 (1H, bs, NH). Anal. calcd for C_9_H_14_NO: C, 71.49; H, 8.67; N, 9.26; Anal. found: C, 71. 30; H, 8.52; N, 9.23. 

### 3.4. Synthesis of Racemic N-Hydroymethyl-9-azabicyclo[6.2.0]dec-6-en-10-one [(±)-***4***]

Racemic **3** (3 g, 19.84 mmol) was dissolved in THF (35 mL) followed by adding paraformaldehyde (0.6 g, 19.84 mmol), K_2_CO_3_ (0.17g, 1.19 mmol) and H_2_O (1.1 mL). The solution was sonicated for 4 h, the solvent was evaporated off and the residue was dissolved in ethyl acetate (50 mL). The solution was dried (Na_2_SO_4_) and then concentrated. The residue was recrystallised from *i*Pr_2_O to afford (±)-**4** as a white crystalline product (3.2 g, 89%; m.p. 81–84 °C). 

The ^1^H-NMR (400 MHz, CD_3_OD, 25 °C, TMS) δ (ppm) data for (±)-**4**: 1.45–2.09 (8H, m, 4 × CH_2_), 3.30–3.32 (1H, dd, *J* = 1.89. 5.52 Hz, H-1), 3.33-3.39 (1H, s, OH), 4.60–4.64 (1H, dd, *J* = 7.78, 11,58 Hz, C*H*_2_OH), 4.69–4.71 (1H, m, H-8), 4.85–4.89 (1H, dd, *J* = 6.47, 11,66 Hz, C*H*_2_OH), 5.57–5.60 (2H, m, C*H*C*H*). Anal. calcd. for C_10_H_15_NO_2_: C, 66.27; H, 8.34; N, 7.73; found: C, 66.19; H, 8.44; N, 7.76.

### 3.5. Gram-Scale Resolution of N-hydroxymethyl-9-azabicyclo[6.2.0]dec-6-en-10-one [(±)-***4***] through Acylation

Racemic **4** (1 g, 5.51 mmol) was dissolved in *i*Pr_2_O (40 mL). Lipase PSIM (1.2 g, 30 mg mL^−1^) then VB (6.29 mL, 55.1 mmol), Et_3_N (a few drops) and Na_2_SO_4_ (0.2 g) were added and the mixture was shaken at 30 °C. After 10 min, the enzyme was filtered off at 51% conversion and *i*Pr_2_O was evaporated. The residue was chromatographed on silica (EtOAc:*n*-hexane 7:3) affording unreacted (1*S*,8*R*)-**4** [0.44 g, 44%; [α]_D_^25^ = –142.4 (*c* = 0.45, EtOH); m.p. 79–83 °C (recrystallized from *i*Pr_2_O); *ee* = 96%] and ester (1*R*,8*S*)-**7** (0.63 g, 46%; ${\left[\alpha \right]}_{D}^{25}$ = +39.2 (*c* = 35, EtOH); *ee* = 94%) as a pale-yellow oil.

The ^1^H-NMR (400 MHz, CDCl_3_, 25 °C, TMS) δ (ppm) data for (1*R*,8*S*)-**7**: 0.93–0.97 (3H, t, *J* = 7.4 Hz, CH_3_), 1.41–1.44 (2H, m, CH_2_*CH*_2_CH_3_), 1.57–2.33 (10H, m, *CH*_2_CH_2_CH_3_ and 4 × CH_2_ from ring), 3.31–3.32 (1H, m, H-1), 4.59–4.60 (1H, dd, *J* = 1.58, 3.88 Hz, H-8), 5.15–5.18 (1H, d, *J* = 11.36 Hz, C*H*_2_OCOPr), 5.20–5.23 (1H, d, *J* = 11.36 C*H*_2_OCOPr), 5.49–5.81 (2H, m, C*H*C*H*). Anal. calcd. for C_14_H_21_NO_3_: C, 66.91; H, 8.42; N, 5.57; found: C, 66.83; H, 8.60; N, 5.42. 

The ^1^H-NMR (400 MHz, CDCl_3_, 25 °C, TMS) δ (ppm) data for (1*S*,8*R*)-**4** are similar to those for (±)-**4**. Anal. found: C, 66.39; H, 8.24; N, 7.73. 

### 3.6. Gram-Scale Resolution of 9-azabicyclo[6.2.0]dec-6-en-10-one [(±)-***3***] through Hydrolysis

Racemic **3** (0.5 g, 3.3 mmol) was dissolved in *i*Pr_2_O (20 mL). CAL-B (0.6 g, 30 mg mL^−1^) and H_2_O (29.7 μL, 1.65 mmol) were added, and the mixture was shaken in an incubator shaker at 60 °C for 5.5 h. The reaction was stopped by filtering off the enzyme at 50% conversion. The solvent was evaporated off and the residue crystallized to give (1*S*,8*R*)-**3** [240 mg, 48%; ${\left[\alpha \right]}_{D}^{25}$ = −140.6 (*c* = 0.5; EtOH); m.p. 151–153 °C recrystallized from *i*Pr_2_O; *ee* = 99%]. The filtered-off enzyme was washed with distilled water (3 × 15 mL), and the water was evaporated off, yielding crystalline β-amino acid (1*R*,2*S*)-**9** [268 mg, 48%; ${\left[\alpha \right]}_{D}^{25}$ = −17 (*c* = 0.35; H_2_O); 266–288 °C with sublimation (recrystallized from H_2_O/acetone); *ee* = 99%].

The ^1^H-NMR (400 MHz, CD_3_OD, 25 °C, TMS) δ (ppm) data for (1*S*,8*R*)-**3** were similar to those for (±)-**3**. Anal. found: C, 71.33; H, 8.57; N, 9.10.

The ^1^H-NMR (400 MHz, D_2_O) δ (ppm) data for (1*R*,2*S*)-**9**: 1.45–2.24 (8H, m, 4 × CH_2_) 2.86–2.90 (1H, m, H-1) 4.42–4.45 (1H, m, H-2) 5.71–6.03 (2H, m, C*H*C*H*). Anal. calcd. for C_9_H_15_NO_2_: C, 63.88; H, 8.93; N, 8.28; found: C, 63.99; H, 8.84; N, 8.28.

### 3.7. Gram-Scale Resolution of N-hydroxymethyl 9-azabicyclo[6.2.0]dec-6-en-10-one [(±)-***4***] through Hydrolysis

(±)-**4** (250 mg, 1.37 mmol) was dissolved in *i*Pr_2_O (10 mL) followed by the addition of CAL-B (300 mg, 30 mg mL^−1^), benzylamine (150 μL, 1.37 mmol) and H_2_O (12.3 μL, 0.68 mmol) and by shaking the mixture in an incubator shaker at 60 °C for 3 h. The reaction was stopped by filtering off the enzyme at 49% conversion. After solvent evaporation the residue (1*S*,8*R*)-**4** crystallized out [120 mg, 48%; ${\left[\alpha \right]}_{D}^{25}$ = −140.4 (*c* = 0.5; EtOH), m.p. 80–83 °C (recrystallized from *i*Pr_2_O), *ee* = 98%]. The filtered enzyme was washed with distilled H_2_O (3 × 15 mL), and crystalline β-amino acid (1*R*,2*S*)-**9** was isolated after evaporation of H_2_O. [109 mg, 47%; ${\left[\alpha \right]}_{D}^{25}$ = −17.1 (*c* = 0.35; H_2_O), m.p. 264–265 °C (recrystallised from H_2_O/acetone), *ee* > 99%].

The ^1^H-NMR (400 MHz, CD_3_OD, 25 °C, TMS) δ (ppm) data for (1*S*,8*R*)-**4** were similar to those for (±)-**4**. Anal. found: C, 66.18; H, 8.35; N, 7.73.

The ^1^H-NMR (400 MHz, D_2_O) data δ (ppm) for (1*R*,2*S*)-**9** from (±)-**4** were similar to those for (1*R*,2*S*)-**9** from (±)-**3** (4.4.). Anal. found: C, 63.95; H, 9.01; N, 8.25. 

### 3.8. Gram-Scale Resolution of N-hydroxymethyl 9-azabicyclo[6.2.0]dec-4-en-10-one [(±)-***6***] through Hydrolysis

Via the procedure described above, the reaction of racemic **6** (250 mg, 1.37 mmol), benzylamine (150 μL, 1.37 mmol) and H_2_O (12.3 μL, 0.68 mmol) in *i*Pr_2_O (10 mL) in the presence of CAL-B (300 mg, 30 mg mL^−1^) at 60 °C afforded amino acid (1*R*,2*S*)-**10** [108 mg, 47%; ${\left[\alpha \right]}_{D}^{25}$ = +24.9 (*c* = 0.3; H_2_O), lit. [26] = +23.9 (*c* = 0.3; H_2_O), *ee* = 95%; m.p.: 264–265 °C (recrystallized from H_2_O/acetone), lit. [26] 218–220 °C; *ee* = 99%] and unreacted (1*S*,8*R*)-**6**, [115 mg, 46%; ${\left[\alpha \right]}_{D}^{25}$ = −28.7 (*c* = 0.5; EtOH), ${\left[\alpha \right]}_{D}^{25}$ = −28.5 (*c* = 1; MeOH), lit. [4] = −27.0 (*c* = 1; MeOH), *ee* = 97%; m.p. 48–49 °C (recrystallized from *n*-hexane), lit. [4] 48–49 °C; *ee* = 99%] after 3 h.

The ^1^H-NMR (400 MHz, D_2_O) δ (ppm) data for (1*R*,2*S*)-**10**: 1.81–2.58 (8H, m, 4 × CH_2_) 2.79 (1H, m, H-1) 3.70–3.73 (1H, m, H-2) 5.72–5.73 (2H, m, C*H*C*H*). Anal. calcd. for C_9_H_15_NO_2_: C, 63.88; H, 8.93; N, 8.28; found: C, 63.59; H, 8.88; N, 8.18. 

The ^1^H-NMR (400 MHz, CDCl_3_, 25 °C, TMS) data δ (ppm) for (1*S*,8*R*)-**6** were similar to those for (±)-**6**: 1.86–2.21 and 2.37–2.44 (8H, m, 4 × CH_2_), 3.28–3.30 (1H, m, H-1), 3.71 (1H, bs, OH), 3.93–3.96 (1H, m, H-8), 4.45–4.48 (1H, d, *J* = 11.6 Hz, C*H*_2_OH), 4.73–4.79 (1H, d, *J* = 11.6 Hz, C*H*_2_OH), 5.67–5.71 (2H, m, C*H*C*H*). Anal. calcd. for C_10_H_15_NO_2_: C, 66.27; H, 8.34; N, 7.73; found: C, 66.10; H, 8.28; N, 7.66.

### 3.9. Synthesis of (1R,8S)-***8*** and (1S,8R)-***8*** through (1R,8S)-***3*** and (1S,8R)-***3*** from β-lactams (1S,8R)-***4*** and (1R,8S)-***7***

Ester (1*R*,8*S*)-**7** (100 mg, 0.66 mmol) was dissolved in MeOH (10 mL), NH_4_OH (1 mL) was added and the mixture was stirred at room temperature for 6 h. The solvent was evaporated off, the residue chromatographed on silica (EtOAc:*n*-hexane 7:3) providing white crystals of (1*R*,8*S*)-**3** [48 mg, 80%; ${\left[\alpha \right]}_{D}^{25}$ = +147 (*c* = 0.5; EtOH); m.p. 152–153 °C (recrystallised from *i*Pr_2_O); *ee* = 95%].

Similarly, (1*S*,8*R*)-**4** (100 mg, 0.55 mmol) afforded white crystals of (1*S*,8*R*)-**3** [59 mg, 71%; ${\left[\alpha \right]}_{D}^{25}$ = −148.7 (*c* = 0.4; EtOH); m.p. 150–153 °C (recrystallised from *i*Pr_2_O); *ee* = 96%].

The ^1^H-NMR (400 MHz, CD_3_OD, 25 °C, TMS) δ (ppm) data for (1*S*,8*R*)-**3** and (1*R*,8*S*)-**3** were similar to those for (±)-**3**. Anal. found for (1*S*,8*R*)-**3**: C, 71.45; H, 8.57; N, 9.22. Anal. found for (1*R*,8*S*)-**3**: C, 71.55; H, 8.67; N, 9.12. 

Palladium-on-carbon (100 mg) was added to (1*R*,8*S*)-**3** or (1*S*,8*R*)-**3** (100 mg, 0.66 mmol) dissolved in a mixture of MeOH (10 mL) and cyclohexene (1 mL). The mixture was treated at reflux temperature for 3 h, and then the catalyst was filtered off. After evaporation, (1*R*,8*S*)-**8** [51 mg, 51%; ${\left[\alpha \right]}_{D}^{25}$ = +17.7 (*c* = 0.5; CHCl_3_); m.p. 104–106 °C (recrystallized from *i*Pr_2_O); *ee* = 98%] or (1*S*,8*R*)-**8** [47 mg, 47%; ${\left[\alpha \right]}_{D}^{25}$ = −17.1 (*c* = 0.5; CHCl_3_), lit. [25] ${\left[\alpha \right]}_{D}^{25}$ = −18 (*c* = 0.5; CHCl_3_)]; m.p. 105–107 °C, lit. [25] m.p. 108–112 °C; *ee* = 96%] was obtained as white crystalline product. 

The ^1^H-NMR (400 MHz, CD_3_OD, 25 °C, TMS) δ (ppm) data for (1*S*,8*R*)-**8** were similar to those for (1*R*,8*S*)-**8**: 1.30–2.09 (12H, m, 6xCH_2_), 3.03–3.06 (1H, m, H-1), 3.65–3.69 (1H, m, H-8), 5.89 (1H, bs, NH). Anal. found for C_9_H_15_NO, (1*S*,8*R*)-**8**: C, 70.48; H, 9.76; N, 9.14 and for (1*R*,8*S*)-**8**: C, 70.51; H, 9.82; N, 9.08. 

### 3.10. Preparation of (1R,2S)-***11*** and (1S,2R)-***11***

Palladium-on-carbon (60 mg) was added to enantiomer (1*R*,2*S*)-**9** or (1*S*,2*R*)-**9** (100 mg) dissolved in MeOH (20 mL) and H_2_ was bubbled through the system at RT for 6 h. The catalyst was then filtered off and after evaporation white crystalline enantiomers of 2-aminocyclooctane-1-carboxylic acid (1*R*,2*S*)-**11** [69.1 mg, 68%; ${\left[\alpha \right]}_{D}^{25}$ = +19.2 (*c* = 0.4; H_2_O), lit. [25] ${\left[\alpha \right]}_{D}^{25}$ = +17.8 (*c* = 0.4; H_2_O); m.p. 250–252 °C (recrystallized from H_2_O/acetone), lit. [25] m.p. 245–248 °C; *ee* = 99%] and (1*S*,2*R*)-**11** [76.2 mg, 75%; ${\left[\alpha \right]}_{D}^{25}$ = −19 (*c* = 0.33; H_2_O); m.p. 241–245 °C (recrystallized from H_2_O/acetone); *ee* = 99%] were isolated.

The ^1^H-NMR (400 MHz, D_2_O) data δ (ppm) for (1*R*,2*S*)-**11** are similar to those for (1*S*,2*R*)-**11**: 1.50–1.92 (12H, m, 6xCH_2_) 2.78–2.79 (1H, m, H-1) 3.59–3.63 (1H, m, H-2). Anal. calcd. for C_9_H_17_NO_2_: C, 63.14; H, 10.01; N, 8.18; found for (1*R*,2*S*)-**11**: C, 63.20; H, 10.18; N, 8.02; found for (1*S*,2*R*)-**11**: C, 63.14; H, 9.91; N, 8.29. 

### 3.11. Acidic Hydrolyses to *β*-amino Acid Hydrochlorides

When the enantiomeric lactam at issue (0.2 mmol) was treated with 18% aqueous HCl (5 mL) at reflux temperature, the desired amino acid hydrochloride was obtained, as follows: 

(1*R*,2*S*)-**9**·HCl [37 mg, 92%; ${\left[\alpha \right]}_{D}^{25}$ = −17.3 (*c* = 0.35; H_2_O); m.p. 218–220 °C (recrystallised from EtOH/Et_2_O); *ee* = 98%] from 50 mg (1*R*,8*S*)-**7**.

(1*S*,2*R*)-**9**·HCl [35 mg, 86%; ${\left[\alpha \right]}_{D}^{25}$ = +19.6 (*c* = 0.5; H_2_O); m.p. 214–218 °C (recrystallised from EtOH/Et_2_O); *ee* = 99%] from 30 mg (1*S*,8*R*) **3**.

(1*S*,2*R*)-**9**·HCl [31 mg, 76%; ${\left[\alpha \right]}_{D}^{25}$ = +19.6 (*c* = 0.6; H_2_O); m.p. 219–220 °C (recrystallised from EtOH/Et_2_O); *ee* = 99%] from 36 mg (1*S*,8*R*) **4**. 

(1*S*,2*R*)-**10**·HCl [38 mg, 93%; ${\left[\alpha \right]}_{D}^{25}$ = −15.0 (*c* = 0.5; H_2_O), lit. [25] ${\left[\alpha \right]}_{D}^{25}$ = −15.9 (*c* = 0.3; H_2_O; m.p. 200–204 °C (recrystallised from EtOH/Et_2_O *ee* = 97%] from 36 mg (1*S*,8*R*)-**6**. 

The ^1^H-NMR (400 MHz, D_2_O) data δ (ppm) for (1*R*,2*S*)-**9**·HCl are similar to those for (1*S*,2*R*)-**9**·HCl: 1.52–2.31 (8H, m, 4 × CH_2_) 3.21–3.24 (1H, m, H-1) 4.55–4.58 (1H, m, H-2) 5.76–6.14 (2H, m, C*H*C*H*). 

The ^1^H-NMR (400 MHz, D_2_O) data δ (ppm) for (1*S*,2*R*)-**10**·HCl: 1.64–2.33 (8H, m, 4 × CH_2_) 2.91–2.98 (1H, m, H-1) 3.66–3.69 (1H, m, H-2) 5.56–5.63 (2H, m, C*H*C*H*). 

Anal. calcd. for C_9_H_16_ClNO_2_: C, 52.56; H, 7.84; N, 6.81; found for (1*R*,2*S*)-**9**·HCl: C, 52.50; H, 7.68; N, 6.80; found for (1*S*,2*R*)-**9**·HCl from (1*S*,8*R*) **3**: C, 52.68; H, 7.64; N, 6.78; found for (1*S*,2*R*)-**9**·HCl from (1*S*,8*R*)-4: C, 52.56; H, 7.66; N, 6.95; found for (1*S*,2*R*)-**10**·HCl: C, 52.47; H, 7.77; N, 6.62. 

## 4. Conclusions

A highly efficient CAL-B (lipase B from *Candida antarctica*)-catalyzed ring-cleavage reaction (*E* > 200) of 9-azabicyclo[6.2.0]dec-6-en-10-one [(±)-**3**)] with 1 equiv. of H_2_O in *i*Pr_2_O at 60 °C resulted new eight-membered ring-fused β-lactam (1*S*,8*R*)-**3** and β-amino acid (1*R*,2*S*)-**9** (*ee* ≥ 99%). Even more efficient two-step transformation was carried out for the ring cleavage of activated lactams (±)-**4** with 1 equiv. of H_2_O, in the presence of CAL-B using 1 equiv. of benzylamine in *i*Pr_2_O at 60 °C. The ring cleavage of regioisomeric (±)-**6**, carried out under the same conditions resulted unreacted (1*S*,8*R*)-**6**, potential intermediate for the synthesis of enantiomeric anatoxin-*a*. These results underline the importance of substrate engineering, since faster reactions were clearly detected when activated lactams vs. their inactivated counterparts were reacted. Advantages of these reactions are the spontaneous degradation of *N*-hydroxymethyl groups and easy product separation by organic solvent–H_2_O extraction.

An indirect enzymatic method, in view of the synthesis of enantiomeric β-amino acids, has also been devised, through a relatively fast acylation of *N*-hydroxymethyl 9-azabicyclo[6.2.0]dec-6-en-10-one [(±)-**4**)] with 10 equiv. of VB mediated by lipase PSIM (*Burkholderia cepacia*) using a catalytic amount of Et_3_N and Na_2_SO_4_ in *i*Pr_2_O at 30 °C (*E* = 114). This route is somewhat less efficient and, in fact, it is a longer procedure to prepare amino acid enantiomers. However, it ensures the simultaneous preparation of new β-lactam enantiomers [(1*S*,8*R*)-**4** and (1*R*,8*S*)-**7**, and (1*S*,8*R*)-**3** and (1*R*,8*S*)-**3**, respectively].

## Figures and Tables

**Table 1:** Enzyme-catalyzed acylation of (±)-**4** ^a^.

Entry	Enzyme (30 mg mL^−1^)	Acyl Donor (Equiv)	Solvent	Temp. (°C)	R. Time (Min)	Conv. (%)	*ee*_s_^b^ (%)	*ee*_p_^b^ (%)	*E*
1	PSIM	VB (2)	*i*Pr_2_O	-15	120	44	76	96	112
2	PSIM	VB (2)	*i*Pr_2_O	2-3	60	43	70	94	67
3	PSIM	VB (2)	*i*Pr_2_O	30	10	46	77	90	44
4	PSIM	VB (10)	*i*Pr_2_O	30	10	51	87	84	32
5	PSIM	VB (10) + Et_3_N + Na_2_SO_4_	*i*Pr_2_O	30	10 25	45 50	77 91	96 92	114 76
6	PSIM	2,2,2-Trifluoroethyl-butyrate(10)	*i*Pr_2_O	30	20	49	83	86	34
7	PSIM	VA (10)	*i*Pr_2_O	30	10	50	81	82	25
8	PSIM	EtOAc(10)	*i*Pr_2_O	30	240	16	12	62	5
9	PSIM	Ac_2_O (10)	*i*Pr_2_O	30	10	52	87	80	25
10	AK ^c^	VB (10)	*i*Pr_2_O	30	20	49	74	78	17
11	AY ^c^	VB (10)	*i*Pr_2_O	30	240	25	14	42	28
12	CAL-A ^c^	VB (10)	*i*Pr_2_O	30	10	29	12	29	2
14	CAL-B	VB (10)	*i*Pr_2_O	30	50	67	2	1	1
14	PPL	VB (10)	*i*Pr_2_O	30	120	20	23	91	26
15	PSIM	VB (10)	*t*BuOMe	30	10	51	87	85	34
16	PSIM	VB (10)	Toluene	30	5	49	89	93	82
17	PSIM	VB (10)	Acetone	30	60	49	86	89	47

^a^ 0.015 M substrate; ^b^ According to GC (Experimental Section); ^c^ Contains 20% (*w*/*w*) of lipase adsorbed on Celite in the presence of sucrose.

**Table 2:** CAL-B-catalyzed ring cleavage of racemic **3** ^a^, **4** ^b^, **5** ^a^ and **6** ^b^.

Entry	Substrate	R. Time (h)	Conv. ^c^ (%)	*ee*_s_^d^ (%)	*ee*_p_^e^ (%)
1	(±)-3	5	43	75	>99
2	(±)-5	5	46	84	>99
3	(±)-4	3	49	96	>99
4	(±)-6	3	50	>99	98

^a^ 0.015 M substrate, 30 mg mL^−1^ CAL-B, 0.5 equiv. of H_2_O, *i*Pr_2_O, 60 °C; ^b^ 0.015 M substrate, 30 mg mL^−1^ CAL-B, 0.5 equiv. of H_2_O, 1 equiv. of benzylamine, *i*Pr_2_O, 60 °C; ^c^ Calculated from *ee_S_* and *ee_P_*; ^d^ According to GC (Experimental Section); ^e^ Determined by using GC, after double derivatization (DD) [31].

**Table 3:** Preparative-scale resolutions.

				Product				Unreacted substrate			
	Reaction Partner	R. Time (Min)	Conv. ^a^ (%)	Yield (%)	Isomer	*ee_P_* (%)	${\left[\alpha \right]}_{D}^{25}$	Yield (%)	Isomer	*ee_S_* (%)	${\left[\alpha \right]}_{D}^{25}$ (EtOH)
(±)-3 ^b^	H_2_O	330	50	48	(1*R*,2*S*)-9	99 ^c^	−17 ^d^	49	(1*S*,8*R*)-3	99 ^e^	−140.6 ^f^
(±)-4 ^g^	VB	10	51	46	(1*R*,8*S*)-7	94 ^e^	+39.2 ^h^	44	(1*S*,8*R*)-4	96 ^e^	−142.4 ^i^
(±)-4 ^j^	H_2_O	180	49	47	(1*R*,2*S*)-9	>99 ^c^	−17.1 ^d^	48	(1*S*,8*R*)-4	98 ^e^	−140.4 ^f^
(±)-6 ^j^	H_2_O	180	50	47	(1*R*,2*S*)-10	99 ^c^	+24.9 ^k^	46	(1*S*,8*R*)-6	99 ^e^	−28.7 ^l^

^a^ Calculated from *ee_S_* and *ee_P_*; ^b^ 0.015 M substrate, 30 mg mL^−1^ CAL-B, 0.5 equiv. of H_2_O, *i*Pr_2_O, 60 °C; ^c^ Determined by GC after DD (Experimental Section); ^d^ *c* = 0.35; H_2_O; ^e^ Determined by GC (Experimental Section); ^f^ *c* = 0.5; ^g^ 0.015 M substrate, 30 mg mL^−1^ PSIM, 10 equiv. of VB, catalytic amount of Et_3_N and Na_2_SO_4,_ *i*Pr_2_O 30 °C; ^h^ *c* = 0.35; EtOH; ^i^ *c* = 0.45; ^j^ 0.015 M substrate, 30 mg mL^−1^ CAL-B, 0.5 equiv. of H_2_O, 1 equiv. of benzylamine, *i*Pr_2_O, 60 °C; ^k^ *c* = 0.3; H_2_O; ^l^ *c* = 0.5.

**Table 4:** Physical data on enantiomers prepared.

Entry	Enantiomers	*ee* (%)	${\left[\alpha \right]}_{D}^{25}$
1	(1*R*,8*S*) 3 from (1*R*,8*S*)-**7**	95	+147 (*c* = 0.5; EtOH)
2	(1*S*,8*R*) 3 from (1*S*,8*R*)-**4**	96	−148.7 (*c* = 0.4; EtOH)
3	(1*R*,8*S*) 8 from (1*R*,8*S*)-**3**	98	+17.7 (*c* = 0.5; CHCl_3_)
4	(1*S*,8*R*) 8 from (1*S*,8*R*)-**3**	96	−17.1 (*c* = 0.5; CHCl_3_)
5	(1*S*,2*R*)-9·HCl from (1*S*,8*R*) **3**	99	+19.6 (*c* = 0.5; H_2_O)
6	(1*S*,2*R*)-9·HCl from (1*S*,8*R*) **4**	99	+19.6 (*c* = 0.6; H_2_O)
7	(1*R*,2*S*)-9·HCl from (1*R*,8*S*)-**7**	98	−17.3 (*c* = 0.35; H_2_O)
8	(1*S*,2*R*)-10·HCl from (1*S*,8*R*)-**6**	97	−15.0 (*c* = 0.5; H_2_O)
9	(1*R*,2*S*)-11 from (1*R*,2*S*)-**9**	99	+19.2 (*c* = 0.4; H_2_O)
10	(1*S*,2*R*)-11 from (1*S*,2*R*)-**9**	99	−19 (*c* = 0.33; H_2_O)
